# Predictors of uterine rupture in a large sample of women in Senegal and Mali: cross-sectional analysis of QUARITE trial data

**DOI:** 10.1186/s12884-018-2064-y

**Published:** 2018-11-01

**Authors:** Rebecca Delafield, Catherine M. Pirkle, Alexandre Dumont

**Affiliations:** 1 Department of Native Hawaiian Health, John A. Burns School of Medicine, University of Hawaiʻi at Mānoa, 677 Ala Moana Blvd., Suite 1015, Honolulu, HI 96813-5401 USA; 2 Office of Public Health Studies, University of Hawaiʻi at Mānoa, 1960 East-West Road, BioMed T102, Honolulu, HI 96822-2319 USA; 3 Research Institute for Development, Université Paris Descartes, Sorbonne Paris Cité, Research Unit 196 (CEPED), Paris, France

**Keywords:** Uterine rupture, Sub-Saharan Africa, Delivery of health care, Dystocia, Referral and consultation

## Abstract

**Background:**

The purpose of this study was to investigate predictors of uterine rupture in a large sample of sub-Saharan African women. Uterine rupture is rare in high-income countries, but it is more common in low-income settings where health systems are often under-resourced. However, understanding of risk factors contributing to uterine rupture in such settings is limited due to small sample sizes and research rarely considers system and individual-level factors concomitantly.

**Methods:**

Cross-sectional data analysis from the pre-intervention period (Oct. 1, 2007- Oct. 1, 2008) of the QUARITE trial, a large-scale maternal mortality study. This research examines uterine rupture among 84,924 women who delivered in one of 46 referral hospitals in Mali and Senegal. A mixed-effects logistic regression model identified individual and geographical risk factors associated with uterine rupture, accounting for clustering by hospital.

**Results:**

Five hundred sixty-nine incidences of uterine rupture (0.67% of sample) were recorded. Predictors of uterine rupture: grand multiparity defined as > 5 live births (aOR = 7.57, 95%CI; 5.19–11.03), prior cesarean (aOR = 2.02, 95%CI; 1.61–2.54), resides outside hospital region (aOR = 1.90, 95%CI: 1.28–2.81), no prenatal care visits (aOR = 1.80, 95%CI; 1.44–2.25), and birth weight of > 3600 g (aOR = 1.61, 95%CI; 1.30–1.98). Women who were referred and who had an obstructed labor had much higher odds of uterine rupture compared to those who experienced neither (aOR: 46.25, 95%CI; 32.90–65.02).

**Conclusions:**

The results of this large study confirm that the referral system, particularly for women with obstructed labor and increasing parity, is a main determinant of uterine rupture in this context. Improving labor and delivery management at each level of the health system and communication between health care facilities should be a priority to reduce uterine rupture.

**Electronic supplementary material:**

The online version of this article (10.1186/s12884-018-2064-y) contains supplementary material, which is available to authorized users.

## Background

Uterine rupture (UR) is a severe complication in pregnancy that involves tearing of the uterine wall during the course of pregnancy or delivery. UR is associated with a substantially increased risk of maternal and perinatal mortality and morbidity when compared to an uncomplicated delivery [1–3]. While the occurrence of UR is relatively rare, it is more frequent in low-income compared to high-income countries [3–5].

Morbidities resulting from UR include hysterectomy, massive hemorrhage, shock, post-hemorrhagic anemia, vesicovaginal fistula, infection or sepsis, and increased risk of rupture in subsequent pregnancies [3, 5, 6]. In high-income settings, the greatest risk factor is a scarred uterus, typically from a previous cesarean delivery. In contrast, while this association is also observed in low-income settings, risk for UR in these contexts appears largely related to factors such as parity, obstructed labor, induction of labor, use of prostaglandins, and/or breech presentation [2, 3, 7].

Research in sub-Saharan Africa highlights other risk factors for UR including lack of prenatal care, limited access to emergency obstetrical care, delays and/or poor management of care [8–12]. Yet these studies are small, frequently focus on one institution and often fail to adjust for potential confounding variables such as parity or previous cesarean delivery when characterizing health system risk factors for UR [8–11]. Published case reports of UR events provide valuable insights, but may not be applicable to the majority of cases that clinicians and health centers encounter [13–17].

Because of the severe consequences of UR, prevention is paramount. Yet, the rarity of UR makes it difficult to study. Therefore, research investigating the factors contributing to UR using large datasets with quality data collection and abstraction of data from medical records is needed. Such information can benefit clinicians, health systems, and communities that experience morbidity and mortality due to UR by identifying potential points of intervention. The purpose of this study is to investigate predictors of UR in a large sample of sub-Saharan African women.

## Methods

This is a cross-sectional analysis of pre-intervention data from the QUARITE (quality of care, risk management and technology in obstetrics) trial, a cluster-randomized multicenter intervention study conducted in Mali and Senegal [18]. The QUARITE trial is registered on the Current Controlled Trials website under the number ISRCTN46950658. Data collection on all births in the study period took place at 46 public referral hospitals (district, regional, and national/teaching hospitals). For more details on the trial protocol and principal results see Dumont et al., 2009 and Dumont et al., 2013 [18, 19]. The sample includes all women (*N* = 84,924) that delivered at any of the 46 referral hospitals during the pre-intervention period (Oct. 1, 2007 – Oct. 1, 2008) of the trial. The data collection system was based on the World Health Organization (WHO) global health survey of maternal and perinatal health, which included collection of institution level and individual-level data [19, 20]. Data on the women, their pregnancy, labor and delivery were extracted from hospital and medical records into a standard one-page data collection form. Given that data were collected on all women at each participating site, the QUARITE investigators kept the data collection instrument relatively short in order to minimize the burden of the trial on health professionals working at the study sites. Trained midwives collected the data from medical records at each site. National coordinators supervised them and data quality was monitored by random audits [18]. Complete data on UR, our principal measure of interest, were available for 84,802 women, or 99.9% of the total number of women who delivered. Records with missing values for UR (*n* = 122) were excluded from the analysis.

The outcome of interest is UR, diagnosed by a health professional. The definition used for the study was, “occurrence of clinical symptoms (pain, fetal distress, acute loss of contractions, hemorrhage) or intrauterine fetal death that lead to laparotomy, at which the diagnosis of uterine rupture was confirmed; or laparotomy for UR after vaginal birth” ([21] p6]). UR has been defined similarly in previous studies [4]. UR was captured as a dichotomous variable (yes/no) in this dataset, no anatomic details of the rupture (e.g., total rupture versus dehiscence) were considered for this outcome. The literature identifies multiple risk factors for UR that fall into four categories: maternal characteristics, obstetric, institutional, and geographical factors [3, 8–12]. Maternal characteristics include a woman’s age at delivery (categorized into < 20, 20–35, > 35 years), and parity for the current delivery (categorized by quartiles into < 1 birth, 2 births, 3–4 births, 5 or more births). The following obstetric factors were included as dichotomous variables: induction of labor, prior cesarean delivery, comorbidity, obstructed labor (defined as slow or arrest of dilation despite ruptured membranes and oxytocin augmentation, non-engagement of presentation at full cervical dilation, or failed vacuum or forceps at full cervical dilation with engaged head), pre-eclampsia/eclampsia, hemorrhage, and > 90th centile of birth weight (> 3600 g) in our sample. Comorbidity was defined as having one or more co-occurring diseases or conditions such as HIV/AIDS, malaria, heart or kidney disease, chronic respiratory condition, gestational diabetes, etc., recorded on the study data collection form. For additional details please see supplementary file (Additional file 1). Mode of delivery was also collected and classified as either spontaneous vaginal delivery, cesarean delivery, and instrumental (forceps or vacuum), breech, or other. Other refers to specific obstetric maneuvers or surgical procedures (e.g., craniotomy) to achieve delivery for obstetric complications, such as fetal malformation with intrauterine fetal death, or transverse presentation or twin retention, etc. These cases may require specific obstetric maneuvers or surgical procedures (e.g., craniotomy) to achieve delivery. The number of prenatal visits (coded as none, 1–4, > 4) was also included in this analysis. The categorization of prenatal care is based on WHO guidelines for prenatal care prior to the November 2016 change, which doubled the minimum recommended prenatal care consultations to eight [22].

Institutional aspects are resources that may or may not be available at each site and included a blood bank, an adult intensive care unit, an anesthesiologist (or staff member trained in anesthesia) on call 24 h a day, and an obstetrician-gynecologist on staff (available for deliveries), each coded as dichotomous variables. Note that the institutional characteristics are not direct risk factors for UR, but serve as proxies for the level of resources available at the hospital for the care of patients. Human resources such as obstetricians-gynecologists and specialized care resources (e.g., blood bank) are associated with higher-level care facilities, but care levels are not strictly dictated by the hospital type. For example, regional hospitals can be either care level one or level two (higher-level) facilities. Higher-level facilities are presumed to provide better quality of care to patients, especially those with more complicated obstetrical conditions [19]. Geographical factors were examined to understand better the influence of geographic/spatial context and accessibility to the referral centers on UR. Previous research has indicated that disparities in mortality between rural and urban settings and delays in care are associated with transport to hospitals [23–25]. Geographical factors in this study include the country where the delivery took place (Senegal or Mali) and the type: a hospital within the capital, a regional hospital outside the capital, or a district hospital. A third geographical factor is the woman’s place of residence in relation to the hospital where she delivered, categorized as: within the same town as the hospital, outside the town but within the region where the hospital is located, or outside of the region where the hospital is located. Referral from another health center, such as a community health center, which provides primary health care, (versus self-referred) is also included in this category. Ambulances will typically provide transportation for women referred from one health center to a higher-level referral hospital (a district, regional, or national/teaching hospital) [24]. An interaction term was created to combine obstructed labor (yes/no) and referred to a referral hospital (yes/no) in the final model due to the strong association between obstructed labor and referral.

Data were analyzed using STATA version 14.0 (STATA Corporation, College Texas USA). A Chi-squared test compared those who experienced UR and those who did not by each covariate individually. A *P* value of < 0.05 was considered statistically significant. A multiple step process tested the independent associations of each variable with UR. In cluster-randomized control trials it is assumed that the similarity or “clustering” of characteristics at any given institution will have some influence on the outcomes of each individual interacting with that specific institution. To account for the clustering of variables by institution, a mixed-effects logistic regression model assuming a random intercept was used in the analysis. A backwards elimination process was conducted to arrive at the most parsimonious model for presenting adjusted odds ratios and confidence intervals for predictors of UR. The first step was to run the full model that included each of the variables from all categories. Based on a cut-off point of a *P* < 0.20, the variable with the highest *P* value over 0.20 was excluded from the model and the model was run again. This step was repeated until all variables were below the cut-off point. Deletions occurred in the following order to get to the final model: adult intensive care unit, anesthesiologist on call, comorbidity, and blood bank. Excluded variables were then re-entered individually to assess the stability of the model. No meaningful changes were observed. Because of their conceptual importance, age and country were retained in the final model, even though their *P* values exceeded the cut-off.

## Results

Among the 84,802 women in our sample for which we had full data, 569 (0.67%) experienced UR. The mean age of women in the study was 26 (range:10–56 years). The mean parity was three with a maximum of 18 births. Among the 569 women who experienced UR, 69 (12%) died. Nearly all of these deaths (62/69) were due to UR or the consequences of UR (i.e., hemorrhage). Other causes of death among those with UR included; eclampsia, infection and unspecified. Table 1 presents the frequency and percent of UR according to maternal and obstetric factors. Most of the factors identified by the literature as likely to be associated with UR were statistically significant (*P* < 0.05) in this sample, based on the bivariate analyses. The exceptions include induction and preeclampsia/eclampsia. While hemorrhage was associated with UR in this analysis, there was no information recorded on whether the hemorrhage occurred prior to the rupture and it is likely that the hemorrhage was a result of the rupture.

**Table 1:** Maternal characteristics and obstetric factors for sample and by uterine rupture

		Total sample		No uterine rupture		Experienced uterine rupture ^a, b^		
		*N* = 84,924 (100%)		*N* = 84,233 (99.19%)		*N* = 569 (0.67%)		
		n	%	n	%	n	%	*p* value
*Maternal characteristics*								
Age	< 20	16,526	19.46	16,458	99.59	45	0.27	< 0.001
	20–35	58,998	69.47	58,515	99.18	408	0.69	
	> 35	8551	10.07	8427	98.55	106	1.24	
Parity ^c^	< 1birth	29,502	34.74	29,418	99.72	55	0.16	< 0.001
	2 births	16,264	19.15	16,178	99.47	72	0.44	
	3 or 4 births	20,133	23.71	19,966	99.17	147	0.73	
	> 5 births	18,977	22.35	18,647	98.26	294	1.55	
*Obstetric factors*								
Mode of delivery	Vaginal	64,993	76.53	64,942	99.92	42	0.06	< 0.001
	Cesarean	16,218	19.1	15,736	97.03	477	2.94	
	Instrumental	1775	2.09	1767	99.55	8	0.45	
	Breech	1544	1.82	1539	99.68	4	0.26	
	Other	91	0.11	59	64.84	32	35.16	
Delivery induced	Yes	2484	2.92	2464	99.19	19	0.77	0.545
	No	82,162	96.75	81,598	99.31	546	0.66	
No. prenatal visits	None	8756	10.31	8592	98.13	135	1.55	< 0.001
	1 to 4	65,024	76.57	64,600	99.35	381	0.59	
	> 4	10,470	12.33	10,421	99.53	44	0.42	
Prior cesarean	Yes	5894	6.94	5771	97.91	121	2.05	< 0.001
	No	78,926	92.94	78,395	99.33	447	0.57	
Comorbidity	Yes	20,384	24.00	20,141	98.81	184	0.90	< 0.001
	No	64,502	75.95	64,086	99.36	385	0.60	
Obstructed labor	Yes	13,822	16.28	13,382	96.82	438	3.17	< 0.001
	No	70,978	83.58	70,841	99.81	130	0.18	
Pre/eclampsia	Yes	2967	3.52	2967	99.20	19	0.64	0.812
	No	81,228	96.31	81,228	99.32	550	0.67	
Hemorrhage	Yes	5220	6.15	4827	92.47	382	7.32	< 0.001
	No	79,594	93.72	79,398	99.75	186	0.23	
> 90% centile of	Yes	9919	11.68	9777	98.57	140	1.41	< 0.001
birth weights	No	74,529	87.76	74,097	99.42	420	0.56	

^a^Data on Uterine Rupture (yes/no) is missing for 122 women or 0.14% of the sample

^b^Missing values (of those that experienced uterine rupture): Age(*n* = 10), Parity (*n* = 1), No. prenatal visits (*n* = 9), Prior cesarean delivery (*n* = 1), Mode of delivery (*n* = 6), Delivery induced (*n* = 4), Breech presentation (*n* = 6), Obstructed labor (*n* = 1), Pre/eclampsia (*n* = 5), ≥ 90th centile birth weight [3600 g] (*n* = 9), Hemorrhage (*n* = 1)

^c^Parity was categorized as ≤ 1 because there were eight deaths of women prior to delivery among the total sample. Among women who experienced UR, there were no nulliparous women

Table 2 presents the frequency and percent of UR according to geographical and institutional characteristics. At an institutional level, UR was less frequent when an anesthesiologist or obstetrician were available and more frequent in facilities with a blood bank or an ICU. All the geographical characteristics were significantly associated with UR in the bivariate analysis.

**Table 2:** Geographical & institutional characteristics for sample and by uterine rupture

		Total sample		No uterine rupture		Experienced uterine rupture ^a, b^		
		*N* = 84,924 (100%)		*N* = 84,233 (99.19%)		*N* = 569 (0.67%)		
		n	%	n	%	n	%	*p* value
*Geographical characteristics*								
Country	Senegal	45,687	53.80	45,338	99.24	278	0.61	0.018
	Mali	39,237	46.20	38,895	99.13	291	0.74	
Hospital type	Capital	37,247	43.86	37,117	99.65	121	0.32	< 0.001
	Regional	29,211	34.40	28,835	98.71	293	1.01	
	District	18,466	21.74	18,281	99.00	155	0.84	
Location of residence	In town of hospital	72,339	85.18	71,867	99.35	433	0.60	< 0.001
	Outside town, same region	9550	11.25	9451	98.96	76	0.80	
	Outside of region	2751	3.24	2683	97.53	56	2.04	
Referral	Yes	21,028	24.76	20,526	97.61	424	2.02	< 0.001
	No	63,871	75.21	63,689	99.72	144	0.23	
*Institutional characteristics*								
Blood bank	Yes	43,695	51.45	43,181	98.82	410	0.94	< 0.001
	No	41,229	48.55	41,052	99.57	159	0.39	
Adult intensive care	Yes	31,468	37.05	31,123	98.90	268	0.85	< 0.001
	No	53,456	62.95	53,110	99.35	301	0.56	
24-h a day anesthesiologist	Yes	45,742	53.86	45,417	99.29	275	0.60	< 0.001
	No	39,182	46.14	38,816	99.07	294	0.75	
Obstetrician	Yes	73,744	86.84	73,190	99.25	457	0.62	< 0.001
	No	11,180	13.16	11,043	99.77	112	1.00	

^a^Data on Uterine Rupture (yes/no) is missing for 122 women or 0.14% of the sample

^b^Missing values (of those that experienced uterine rupture): Location of residence (*n* = 4), Referral (*n* = 1)

Table 3 presents the results of the multivariate analyses. The interaction term of obstructed labor and referral showed a strong and clear relationship between obstruction, referral, and the likelihood of UR. Of those referred, 41.25% were diagnosed with obstructed labor (not shown). The odds of UR clearly increased as parity increased and were highest for women that had a parity of five or more births compared to women with lower parity even after adjusting for previous cesarean delivery. It should be noted that there were no URs among nulliparous women, so data in the lowest parity category represent women with a parity of one for the current pregnancy. Maternal age was not significant in this model either as a categorical variable or as a continuous variable (not shown).

**Table 3:** Mixed-effects logistic regression analysis of predictors for uterine rupture^a^

		n	%^b^	aOR	SE	95%CI	*p* value
*Geographical characteristics*							
Country	Senegal	278	48.86	Ref			
	Mali	291	51.14	1.33	0.24	0.94–1.89	0.112
Hospital Type	Capital	121	21.27	Ref			
	Regional	293	51.49	1.59	0.32	1.08–2.34	0.018
	District	155	27.24	1.22	0.24	0.82–1.80	0.327
Location of residence	In town of hospital	433	76.10	Ref			
	Out of town, same region	76	13.36	1.06	0.19	0.75–1.50	0.736
	Outside of region	56	9.84	1.90	0.38	1.28–2.81	0.001
*Maternal characteristics*							
Age	< 20	45	7.91	1.06	0.21	0.71–1.56	0.789
	20–35	408	71.70	Ref			
	> 35	106	18.63	1.09	0.13	0.85–1.38	0.498
Parity^c^	1 birth	55	9.67	Ref			
	2 births	72	12.65	2.74	0.56	1.84–4.07	< 0.001
	3 or 4 births	147	25.83	4.89	0.96	3.33–7.19	< 0.001
	> 5 births	294	51.67	7.57	1.45	5.19–11.03	< 0.001
*Obstetric factors*							
No. Prenatal Visits	None	135	23.73	1.80	0.21	1.44–2.25	< 0.001
	1 to 4	381	66.96	Ref			
	> 4	44	7.73	0.98	0.17	0.70–1.36	0.891
Prior Cesarean	Yes	121	21.27	2.02	0.23	1.61–2.54	< 0.001
	No	447	78.56	Ref			
Obstructed labor + Referral	Not obstructed or referred	43	7.56	Ref			
	Not obstructed, Referred	87	15.29	7.61	1.50	5.17–11.19	< 0.001
	Obstructed, Not referred	101	17.75	23.65	4.57	16.20–34.53	< 0.001
	Obstructed + Referred	336	59.05	46.25	8.04	32.90–65.02	< 0.001
> 90th centile birth weight	Yes	140	0.25	1.61	0.17	1.30–1.98	< 0.001
	No	420	0.75	Ref			

^a^Missing values: Age (*n* = 10), Parity (*n* = 1), No. Prenatal Visits (*n* = 9), Referral (*n* = 1), Prior cesarean delivery (*n* = 1), Obstructed labor (*n* = 1), ≥ 90th centile birth weight [3600 g] (*n* = 9), Location of residence (*n* = 4)

^b^Percentage of uterine rupture cases

^c^Among cases of uterine rupture all women in the lowest parity category were parity = 1 representing the current pregnancy

## Discussion

UR in this large sample was significantly influenced by multiple factors which corroborate maternal and obstetrical risk factors identified in smaller studies [9–12]. An important contribution of this study is the pronounced relationship between obstructed labor and referral to a referral hospital. This finding, in conjunction with the independent association between UR and birth weight > 3600 g raises concerns about quality of care. Specifically, concerns about the timing and accuracy of the diagnosis and management of obstructed labor, as well as concern about referral systems and timely transportation to referral hospitals. Calls for improving quality of care for maternal and child health are not new but should be heeded to prevent the high levels of maternal morbidity and mortality in sub-Saharan Africa and elsewhere [26]. Interventions that address these system issues, particularly improvement in the management of labor and referral processes for women with obstructed labor, have the potential to improve the effectiveness of emergency obstetric interventions at the level of a referral hospital.

The higher odds of UR at regional compared to district hospitals (with fewer resources to manage obstetric emergencies) is likely due to selection processes with high-risk cases being referred to regional facilities. While regional referral hospitals have resources to perform interventions that may prevent UR (e.g., cesarean delivery), our work suggests that the usefulness of certain institutional level interventions could be constrained by other factors. Delay in care is a significant contributor to maternal mortality and morbidity [3, 23–25]. This analysis cannot provide details on specific sources of delay, but results suggest that delay in seeking care, delay in decision to refer for obstructed labor (which may indicate poor labor and delivery management) or delay in transportation to a referral hospital likely impact this outcome. With regard to transportation, previous studies have noted poor roads as well as long distances and lack of available transport as obstacles to care [24, 25].

Previous work in Western Mali examined in-hospital maternal mortality by time traveled for women who underwent a cesarean section and those who did not. The authors observed that among women who traveled 4 h or more, case-fatality was dramatically higher among women who underwent a cesarean delivery compared to those who did not [24]. The authors noted that these women likely arrived at the hospital beyond the point at which the cesarean delivery was an effective intervention. Instrumental delivery (forceps or vacuum) is another intervention that could prevent UR [27]. Research conducted at a busy referral hospital in Uganda found a decrease in UR with increased use of vacuum extraction [27]. Instrumental delivery was used in only 2% of deliveries in our study. It is possible that interventions to increase appropriate use of vacuum extraction may help to reduce the incidence of uterine rupture in this context, similar to what was found in the intervention study in Uganda [27]. Based on our work and other studies in sub-Saharan Africa, it appears that even when resources for obstetric emergencies are available at referral hospitals, often interventions are provided too late for many women with obstructed labor, leading to UR [24, 27].

The association of UR with birth weight > 3600 g suggests problems with cephalopelvic disproportion (a risk factor for obstructed labor). Neither breech presentation nor induction, risk factors identified in previous work, were significantly associated with UR in our study [3]. Because breech and induction are potential contributors to obstructed labor, an independent association may have been masked by the strong association between obstructed labor and UR.

Another important finding is the clear pattern of increased odds of UR with increasing parity after controlling for covariates. Other authors have found an increase in UR with higher parity [3, 7, 9]. For example, a study examining risk of complete UR in Norway found women (without a previous cesarean delivery) with a parity of three or more had 2.4 greater odds of complete UR compared to women with less than three births [7]. Results on the influence of grand multiparity compared to lower parity (2–4 births) on UR from studies in low-income countries are mixed [3]. This may be due to smaller sample sizes and lack of statistical control for covariates. Our study adds to the literature by capturing the increase in risk for UR at more discrete levels of parity and controlling for covariates such as previous cesarean delivery. The higher odds of UR as the number of deliveries increases may be due to weakening of the uterus or factors not captured in this study [3]. Based on these findings, interventions to address unmet contraceptive needs in multiparous women and/or programs that support multiparous women with parity greater than three to deliver at a hospital may help to reduce UR in these communities.

Lack of prenatal care was associated with increased odds of UR. As others have noted, the absence of prenatal care suggests women at greater risk for UR are not engaging with the health system, which may impact their decisions about when to seek care at the point of childbirth [3, 25]. Improved access/utilization of the health care system during the prenatal and pre/interconception period could help reduce UR in this sample. More research at the community and institution levels could add to our understanding about how obstructed labor is identified and addressed, how referrals and transportation to referral centers are managed, and how decisions to refer or to seek services from the health care system are made.

This study has several strengths. The enrolled institutions represent 94% of all referral hospitals in Mali and Senegal; therefore, this work may be generalizable to other countries in the region with similar health care systems. The data collection and abstraction were audited for completeness as part of the QUARITE protocol, which minimized problems due to missing data and concerns about the data quality. The large sample size allowed evaluation of associations between variables while controlling for multiple covariates, which may not be feasible in other studies.

Limitations should also be noted. Because the primary focus of the QUARITE trial was maternal mortality, certain variables potentially pertinent to UR were absent (e.g. interconception intervals, quantification of the number of previous cesarean deliveries, exposure to trauma, or scarring of the uterus due to reasons other than prior cesarean delivery). Also, while this study is likely to be representative of factors that impact deliveries in the referral hospitals, a significant proportion of births in Mali and Senegal take place at home (43.4% and 27% respectively); therefore, our results may not be generalizable beyond the scope of referral hospitals [28, 29].

## Conclusions

The results of this large study confirm the strong influence of the referral system as a major determinant of UR, particularly for women with obstructed labor in Senegal and Mali. This work also provides evidence of a dose-response relationship between parity and the odds of experiencing UR in this context. Improving labor and delivery management at each level of the health system and communication between health care facilities should be a priority to reduce UR. Furthermore, efforts to improve interventions upstream from delivery such as addressing unmet needs in family planning and increasing access and engagement in prenatal care could help decrease the risk of UR for women in such settings.

## Additional file

Additional file 1: List of comorbidities. List of any comorbidity included in the dichotomous variable “Comorbidity.” If coded “yes” the individual had one or more of the comorbidities listed (DOCX 17 kb)

## Abbreviations

aOR: Adjusted odds ratio
CI: Confidence interval
QUARITE: Quality of care, risk management and technology in obstetrics
SE: Standard error
UR: Uterine rupture
