# Supplementary material for: Predictors of uterine rupture in a large sample of women in Senegal and Mali: cross-sectional analysis of QUARITE trial data
Source: BMC Pregnancy Childbirth. 2018 Nov 1;18:432. doi: 10.1186/s12884-018-2064-y (PMC6211600; doi:10.1186/s12884-018-2064-y)
Supplement: Supplementary file 1 — List of comorbidities. List of any comorbidity included in the dichotomous variable “Comorbidity.” If coded “yes” the individual had one or more of the comorbidities listed (DOCX 17 kb) [file 12884_2018_2064_MOESM1_ESM.docx]

**Additional file 1.**

| **List of Comorbidities** | |
| --- | --- |
| 1 | Premature rupture of the membranes/ draining of vaginal fluid |
| 2 | Chorioamnionitis or intraamniotic infection |
| 3 | Preterm labor or risk of preterm labor |
| 4 | Gestational hypertension |
| 5 | Chronic hypertension |
| 6 | Pre-eclampsia; HELPP syndrome |
| 7 | Eclampsia; eclamptic crises; seizures prior to delivery |
| 8 | Heart or kidney disease |
| 9 | Chronic respiratory disease |
| 10 | Insufficient fundal height for gestational age or intrauterine growth restriction |
| 11 | Intrauterine fetal death |
| 12 | Post-term or prolonged pregnancy |
| 13 | Gestation diabetes |
| 14 | Malaria |
| 15 | Sickle cell disease |
| 16 | Severe anemia (hemoglobin less than 7g/l) |
| 17 | End of pregnancy vaginal bleeding |
| 18 | Pyelonephritis /urinary infection |
| 19 | Cholestasis |
| 20 | Viral hepatitis |
| 21 | TB |
| 22 | HIV/AIDS |
| 23 | Excessive fundal height; excessive uterine volume |
| 24 | Macrosomia |
| 25 | Restricted pelvis; small pelvis; asymmetric pelvis; contracted pelvis |
| 26 | Placentia previa |
| 27 | Vaginal or perineal warts (genital warts) |
| 28 | Measles |
| 29 | Hydramnios or excessive amniotic fluid |
| 30 | Insufficient amniotic fluid |
| 31 | Syphilis |
| 32 | Genital infections |
| 33 | Typhoid fever |
| 34 | Other pathology |
